# APPLYING TIME-VARYING EFFECT MODELING TO EXAMINE DAILY VARIATION IN SUICIDAL IDEATION AMONG DEMENTIA CAREGIVERS

**DOI:** 10.1093/geroni/igad104.2217

**Published:** 2023-12-21

**Authors:** Frank Puga, Abigail Poe, Danny Wang

**Affiliations:** University of Alabama at Birmingham, Birmingham, Alabama, United States; University of Alabama at Birmingham, Birmingham, Alabama, United States; The Pennsylvania State University, University Park, Pennsylvania, United States

## Abstract

Emerging evidence suggests that caregivers may experience high rates of suicidal ideation. However, relatively little is known about daily contextual factors, including day-to-day depression and anxiety-related symptoms, that increase the severity of suicidal ideation over time. The purpose of this study was to apply time-varying effect modeling (TVEM) to examine daily depression and anxiety symptoms leading up to an individual’s highest suicidal ideation score within a span of 14 days in a sample of community-dwelling ADRD caregivers. The intercept-only model revealed an increase in the mean scores of daily suicidal ideation four days leading up to an individual’s highest score. Additionally, there was a significant decrease four days after the highest suicidal ideation point. The relationship between depression and daily suicidal ideation was significant 1-day leading up to the highest suicidal ideation score. Similar relationships were observed with daily anxiety as a time-varying predictor in a separate model. The results from this study suggest that suicidal ideation can vary between days, with depression and anxiety influencing the intensity of the slope leading to and following an individual’s highest suicidal ideation score. Examining suicidal ideation trajectories over time and factors associated with varying severity can help inform targeted interventions to support dementia caregivers and mitigate the risk of severe psychological distress associated with dementia caregiving.

